# Development of a Recombinase Polymerase Amplification Assay for Detection of Epidemic Human Noroviruses

**DOI:** 10.1038/srep40244

**Published:** 2017-01-09

**Authors:** Matthew D. Moore, Lee-Ann Jaykus

**Affiliations:** 1 Department of Food, Bioprocessing and Nutrition Sciences, North Carolina State University, 315 Schaub Hall, 400 Dan Allen Drive, Raleigh, North Carolina 27695, United States of America

## Abstract

Human norovirus is a leading cause of viral gastroenteritis worldwide. Rapid detection could facilitate control, however widespread point-of-care testing is infrequently done due to the lack of robust and portable methods. Recombinase polymerase amplification (RPA) is a novel isothermal method which rapidly amplifies and detects nucleic acids using a simple device in near real-time. An RT-RPA assay targeting a recent epidemic human norovirus strain (GII.4 New Orleans) was developed and evaluated in this study. The assay successfully detected purified norovirus RNA from multiple patient outbreak isolates and had a limit of detection of 3.40 ± 0.20 log_10_ genomic copies (LGC), which is comparable to most other reported isothermal norovirus amplification methods. The assay also detected norovirus in directly boiled stool, and displayed better resistance to inhibitors than a commonly used RT-qPCR assay. The assay was specific, as it did not amplify genomes from 9 non-related enteric viruses and bacteria. The assay detected norovirus in some samples in as little as 6 min, and the entire detection process can be performed in less than 30 min. The reported RT-RPA method shows promise for sensitive point-of-care detection of epidemic human norovirus, and is the fastest human norovirus amplification method to date.

Human norovirus is estimated to account for a fifth of all acute gastroenteritis cases worldwide^1^, costing $2.8–$3.7 billion in annual economic losses in the U.S. alone^2^. Norovirus is especially troublesome in healthcare settings, as outbreaks result in consumption of resources, extended hospital stays, ward closures, and high morbidity^3^. Thus, early detection of clinical infection is important as it can facilitate more rapid implementation of rigorous controls, which can result in reduced health care costs and improved public health^4^.

Detection of human norovirus historically relies on reverse transcriptase quantitative polymerase chain reaction (RT-qPCR). However, this method requires time-consuming sample preparation and purification and is sensitive to matrix-associated inhibitors^5^. RT-qPCR also relies on bulky instrumentation and usually takes over an hour to complete. Recombinase polymerase amplification (RPA) is a novel isothermal PCR alternative that produces results in 20 minutes or less with portable instrumentation. RPA uses bacterial recombinase enzymes to anneal primers to template DNA for extension and amplification by an isothermal polymerase^6^,^7^. The basic RPA platform has been used in concert with a reverse transcriptase and a fluorescent probe system for real time detection of viral pathogens with RNA genomes^8^,^9^. Its use of sequence repair enzymes theoretically provides a higher fidelity than RT-qPCR^6^,^7^, although the assay’s sensitivity to matrix-associated inhibitors is poorly characterized. The purpose of this study was to develop a real time RT-RPA assay for rapid detection of a recent epidemic human norovirus strain and evaluate its performance in both purified and minimally processed outbreak-derived clinical (stool) specimens.

## Results

### Development and Screening of RT-RPA primer and probe sets

**Figure 1:**
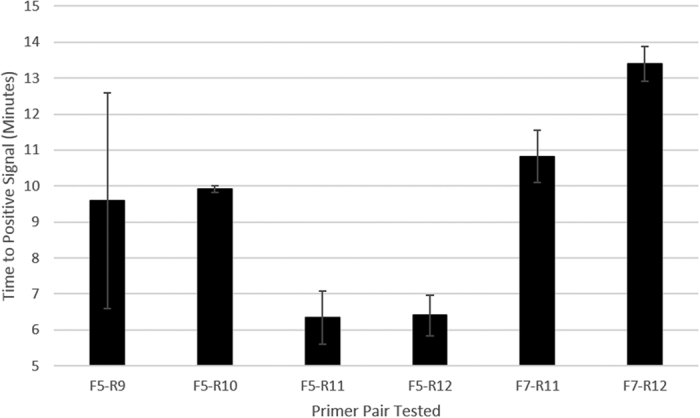
Time to signal detection of different RT-RPA primer pairs. The RT-RPA assay was performed as described using the NOP1 probe. The template used was 7.0 LGC of purified GII.4 New Orleans RNA per reaction.

Forty-eight combinations of candidate primers (8 forward and 12 reverse) were generated and screened for reactivity to purified GII.4 New Orleans RNA. Of these, 8 primer sets were identified as capable of amplifying target RNA, and a probe (NOP1) was designed that would accommodate all 8 sets (Table 1). The primer sets were then tested for the time to fluorescence threshold with the probe and compared (Fig. 1). Six primer sets produced signal with the probe, and two of the sets—NOF5-NOR11 and NOF5-NOR12—produced signal significantly (*p* < 0.05) faster than three other sets and also performed similarly when used on heated stool (data not shown). One set was discarded because it produced signal with widely inconsistent time to result. Due to resource constraints, one set (NOF5-R11 with NOP1) was chosen for subsequent evaluation.

### Evaluation of RT-RPA performance with GII.4 New Orleans outbreak samples

RT-RPA performance was tested using serially diluted stool samples obtained from 12 different individuals during 10 different GII.4 New Orleans outbreaks that occurred in 2012. Two sample preparation methods were analyzed for each of the samples—conventional RNA purification and direct detection after boiling of patient stool (Table 2). RT-RPA applied to serially-diluted purified RNA samples produced signal for a wide range of virus input levels, was highly repeatable, and identified virus in all patient samples. RNA copy number over a range of 0.2 log_10_ genomic copies (LGC) to 9.4 LGC per reaction produced positive signal fluorescence within 6.1 min to 21.5 min. Overall for purified RNA, the input LGC of RNA as determined by RT-qPCR was inversely proportional to the time to signal of the RT-RPA when analyzed by linear regression (Figure S2).

When applied to heated stool, RT-RPA did not perform as well, but positive signal was still repeatedly produced for all outbreak-derived stool samples except one (sample 44), with estimated input genome concentration ranging from 0.8–10.0 LGC and positive signal produced in 6.7–20.0 min. For most boiled stool samples, the RT-RPA produced positive signal for samples with high levels of inhibitors. For example, for relatively “dirty” samples (20% and 2% fecal suspensions), RT-RPA produced more positive replicates than did RT-qPCR, with 61% versus 18% positive replicates for 20% stool, and 61% versus 58% for 2% stool, respectively (Figure S1). Supplementation of reactions with dimethyl sulfoxide (DMSO), formamide, and RNase inhibitor did not improve the results of the RT-RPA assay (Figure S3).

### Analytical sensitivity of RT-RPA for detection of human norovirus GII.4 New Orleans RNA

**Figure 2:**
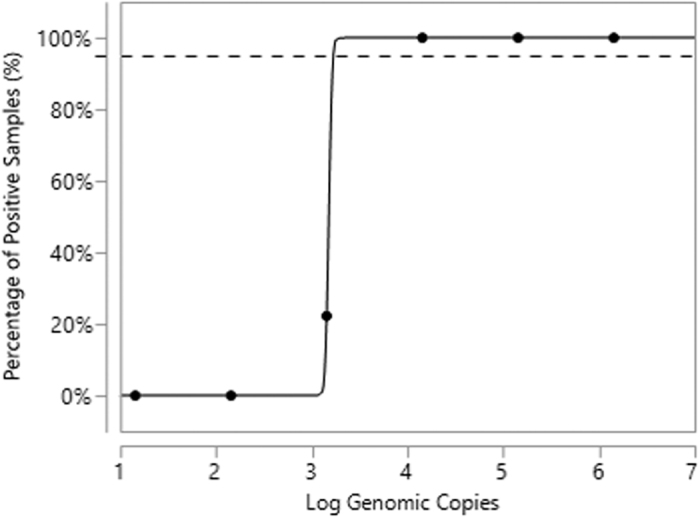
Sensitivity of RT-RPA assay for purified GII.4 New Orleans RNA as predicted using probit regression analyses. Serial dilutions of purified RNA from three selected patient stool samples were used as templates in RT-RPA reactions for 8 replicates each and the number of positive samples at each dilution was used for separate probit regressions. A representative regression analysis (for sample 29) is pictured. The points mark the proportion of samples positive at each log_10_ genomic copy level; the solid line marks the predicted frequency of samples positive as a function of log_10_ genomic copy input, and the dotted line is a visual marker at the 95% level of sample positivity.

To test the analytical sensitivity (detection limit) of the RT-RPA assay, serial dilutions of purified GII.4 New Orleans RNA from three patients (isolates 29, 47, and 58) were tested for 8 replicates and probit regressions used to interpolate a limit of detection (Fig. 2). The range of input RNA producing consistently positive results for all three replicates was 3.2 to 6.2 LGC with time to threshold florescence of 12.4 ± 2.4 min to 8.2 ± 0.5 min, respectively. Based on the probit regressions average, the limit of detection of the assay (the level of input RNA for which 95% of samples would be positive) is 3.40 ± 0.20 LGC. This is about 1.0 LGC higher than the limit of detection determined for RT-qPCR, which was predicted by probit to be 2.3 ± 0.04 LGC.

### Specificity of human norovirus GII.4 New Orleans RT-RPA

The specificity of the RT-RPA was determined using a panel of genomes extracted from relevant enteric virus and bacterial cultures (Table 3). Consistent positive signal was only observed for both GII.4 human norovirus strains. Human norovirus GII.3 produced positive signal for one replicate at one dilution, but was otherwise negative. The GII.4 Sydney strain, the most recent circulating epidemic strain^10^, repeatedly produced positive signal in 8.1–15.3 min over a 3.0 LGC range of input RNA. The repeatable amplification and positive signal of GII.4 Sydney suggests that some degree of base mismatching is tolerated in the RT-RPA system, despite its reliance on recombinase enzymes (Table 4). There were a total of 6 mismatches in the assay’s targeted region when comparing GII.4 New Orleans and Sydney strains, whereas there were 16 mismatches when comparing GII.3 with GII.4 New Orleans. Sydney does not differ from New Orleans in the probe region and has only 4 and 3 base mismatches in the forward and reverse primer target regions, respectively. GII.3 has 3 probe, 2 forward, and 11 reverse primer target region mismatches. It is likely that the reverse primer target region is the reason for the lack of consistent reactivity for GII.3.

## Discussion

In this work, a RT-RPA assay for the rapid detection of GII.4 human norovirus was developed and proof-of-concept data provided for its use in detecting virus in representative clinical samples. In purified RNA extracts, the assay limit of detection was 3.40 LGC per reaction. When human fecal samples positive for norovirus GII.4 New Orleans were extracted for RNA isolation followed by RT-RPA, virus could be detected in all patient specimens, with longer amplification times to target signal corresponding to lower input template concentrations. It was possible to detect norovirus when 20% fecal specimens were heated to release the viral RNA, without further purification. With this heat release method, lower limits of detection were around 5.0 LGC per reaction. Although not as sensitive as the RT-qPCR, the RT-RPA assay appears to have similar analytical sensitivity to conventional RT-PCR and other human norovirus isothermal assays^11^,^12^.

For point-of-care diagnostics in particular, isothermal amplification methods are of great interest due to their convenience, rapid time-to-result, and potential for miniaturization. Two of these methods have been well studied for human norovirus detection: nucleic acid sequence based amplification (NASBA) and loop-mediated isothermal amplification (LAMP). Greene *et al*.^12^ developed the first norovirus NASBA, targeting Norwalk virus, reporting a detection limit of 10^4^ PCR-amplifiable units/ml sample and a total time-to-result of 4–6 h. Moore *et al*.^13^ evaluated the sensitivity and specificity of this same NASBA using GI, GII, and outbreak strains. The method was able to detect 13/17 of the strains tested and, compared to the RT-PCR “gold standard,” had a sensitivity and specificity of 100% and 80%, respectively. A different NASBA assay design targeting the ORF1-ORF2 junction and using the JJV2F and COG2R primers^14^,^15^ reportedly had higher analytical sensitivity, approximately 90% concordance with other assays, and reduced time-to-result (90 min)^16^.

The first human norovirus LAMP assay targeted a similar genome region to the RT-RPA, was designed to detect both GI and GII viruses, and had a detection limit of 2–3 LGC/25 μl reaction. Sensitivity and specificity approached 100% and results were achieved in 60–90 min^11^. Yoda *et al*.^17^ designed their own LAMP assay targeting the ORF1-ORF2 junction and were able to improve on the detection limit (to 1–2 LGC/25 μl reaction) and increase inclusivity by design changes. In another study, Iturriza-Gomara *et al*.^18^ evaluated a commercial RT-LAMP for norovirus detection relative to an RT-PCR, finding a slightly lower sensitivity for the GI RT-LAMP but a higher sensitivity for the RT-LAMP assay. Newer LAMP designs are facilitating colorimetric endpoints and can be used even for genotyping^19^,^20^.

Although several isothermal techniques for the amplification and detection of purified nucleic acids exist^21^,^22^, RPA has multiple advantages, i.e., (i.) the use of a single tube; (ii.) real-time detection of amplified product using a fluorescent probe; (iii.) the inclusion of most required reagents in the form of a freeze-dried pellet to simplify testing; (iv.) the inclusion of a binding protein that has been reported to aid in amplification of RNA targets having a high degree of secondary structure; and (v.) a reduced time to a positive signal. A disadvantage with respect to human norovirus detection may be lack of broad reactivity (i.e., high specificity), perhaps because the recombinase enzyme used is thought to have higher fidelity than PCR^6^,^7^. However, in our work some degree of base mismatch appears to have been tolerated as we were able to detect two different GII.4 strains, although older strains have not been evaluated. However, having a method that reliably detects GII.4 norovirus is relevant as this genotype is responsible for at least 70% of norovirus cases in the United States^23^ and 80–95% of outbreaks globally^24^. New RT-RPA assays could be redesigned relatively quickly to cover new epidemic strains as they emerge.

With the exception of the early work of Greene *et al*.^12^, this is the first study of isothermal amplification in which fecal suspensions without prior RNA extraction were used as template. Our results are generally consistent with Greene *et al*.^12^ in that the RT-RPA assay produced more positives in samples with higher concentrations of fecal material as compared to RT-qPCR. Specifically, for our study’s comparisons of sample positivity for 20% stool suspensions, about 60% were positive by RT-RPA while less than 20% of those same samples were positive by RT-qPCR (Figure S1). This suggests that the RT-RPA method may have more tolerance for inhibitory compounds. When RPA was used for the direct detection of *Chlamydia trachomatis* from urine samples, no notable amplification inhibition occurred, although it was common when using PCR^25^. The authors hypothesized that RPA would be less susceptible to amplification inhibition because the assay relies on enzyme components and conditions more comparable to biological systems. For example, the use of a recombinase enzyme may provide increased primer binding efficiency. A polymerase derived from a bacterial species (*Bacillus subtilis*) that can grow at 37 °C may be more physiologically familiar, as would be an amplification reaction occurring under isothermal conditions of 40 °C^7^. Interestingly, the isothermal LAMP assay has also been shown to have a higher tolerance for inhibitors in other biological samples^26^,^27^,^28^. For instance, Enotomoto *et al*.^28^ successfully directly detected herpes simplex virus-1 (HSV-1) in swab samples from patients with gingiovostomatitis or vesicular skin eruptions. In the same year, Kaneko *et al*.^27^ found similar evidence for HSV-1 and HSV-2 with a LAMP assay considerably outperforming PCR when samples were directly amplified without DNA purification in patients with genitalitis, keratitis, and uveitis. Perhaps the most comprehensive evaluation of the sample matrix on isothermal amplification, Kaneko *et al*.^26^ evaluated the HSV-1 LAMP assay relative to PCR in saline solution, PBS, Modified Eagle’s Medium, serum, plasma, urine, aqueous, and vitreous solutions of different concentrations spiked with a low level of HSV-1 DNA. Although inhibition was inevitably noted at high concentrations for both assays, LAMP outperformed PCR^26^. However, further comparisons of isothermal methods to PCR are necessary before broad conclusions about the impact of inhibitors on amplification efficiency can be made.

This GII.4 norovirus RT-RPA assay displayed poorer analytical sensitivity (detection limit) than several previously reported assays for other viral pathogens. These earlier studies used an RNA standard to determine analytical sensitivity and showed RT-RPA detection limits <1 LGC for bovine coronavirus^9^, and Rift Valley fever, Ebola, Marburg, Sudan and Sigma viruses^29^,^30^. This is 2 LGC better than the limit of detection reported for our assay. However, our limit of detection was similar to that reported for a foot and mouth disease virus assay (3.16 LGC)^31^. There are a number of explanations for the differences in detection limits reported in the literature. Firstly, in the earlier studies, detection limits were based on an RNA standard generated from a plasmid, while our study used RNA directly purified from clinical samples. We believe that the latter approach, while producing higher detection limits, is more biologically relevant, because a full length genome may consume more RPA enzyme and also contain regions of increased secondary structure, both of which can impede enzymatic action^32^. Alternatively, the length of the amplicon reported here is longer than those reported by Euler *et al*.^29^,^30^ and Amer *et al*.^9^, but is similar in length to that reported by Abd El Wahed *et al*.^31^, who showed a similar higher limit of detection.

The RT-RPA assay was able to amplify the two most recent circulating epidemic GII.4 norovirus strains. This implies that despite its high fidelity, the assay is able to tolerate slight differences in target sequence. This is in agreement with Boyle *et al*.^33^, who reported that two different HIV-1 RPA primer-probe sets were capable of amplifying nearly all HIV-1 subtypes, including one isolate with 9 mismatches. In our report, GII.4 Sydney containing 6 total mismatches in the target region was readily amplified. The data of Daher *et al*.^34^, which focused on using RPA for amplification of conserved genes for several bacteria, showed a similar tolerance to base pair mismatches. Interestingly, the GII.3 template was inconsistently amplified by the NOF5-NOR11-NOP1 primer-probe set, the amplified region of which contained base mismatches on the 3′ end of NOF5 and NOR11 target regions in addition to a large number of mismatches (Table 4). Such placement of mismatches was found by Daher *et al*.^34^ to be more inhibitory to amplification than internal or 5′ mismatches. The fact that the primer-probe set reported here did not amplify any other relevant enteric virus or bacterial nucleic acid suggests that it shows good specificity, reducing the likelihood of false positives. However, future studies building upon the foundational work presented here analyzing a larger number and diversity of human norovirus clinical samples must be completed to validate and evaluate the suitability of this assay for large scale clinical settings. For instance, analysis of the assay’s reactivity with earlier strains of the GII.4 genotype and reactivity with the GII.17 genotype that is emerging as a genotype of significance^35^ would be valuable future work. Another logical future direction to build on this work would be to design and optimize more degenerate primer-probe sets and potentially multiplex the assay for immediate genogrouping analysis.

This is the first report on the use of the emerging RPA technology for the rapid detection of human norovirus. RT-RPA has multiple advantages over RT-qPCR, including: (i.) the lack of reliance on larger, more expensive equipment for amplification and detection; (ii.) the use of recombinase enzymes that reduce the likelihood of false positive results due to their inherent proofreading capabilities^6^,^7^,^36^; (iii.) quicker time-to-result; and (iv) the potential for reduced impact of matrix-associated inhibitors. Theoretically, the RT-RPA assay is capable of direct, portable detection of epidemic human norovirus in clinical samples with minimal expertise in less than 30 minutes. With further optimization, in particular improvement of analytical sensitivity and comprehensive evaluation of assay specificity, RT-RPA has potential to be a promising alternative to RT-qPCR or other isothermal methods for rapid testing for human norovirus.

## Methods

### Viruses, Bacteria, and Outbreak Stool Specimens

Twelve stool specimens from human norovirus outbreaks that occurred in 2012 in North Carolina were classified at genogroup II, genotype 4 (GII.4) New Orleans using RT-qPCR and sequencing. Additional stool specimens from outbreaks in 2015 in North Carolina were classified in a similar manner as GII.4 Sydney and GII.3 were obtained for inclusivity/exclusivity testing. These were provided courtesy of S.R. Greene (North Carolina Department of Health and Human Services, Raleigh, NC). Because the samples were provided de-identified, they were exempt from NCSU IRB review, and their use in the study was consistent with institutional biosafety committee certifications. Stool specimens were diluted to 20% (w/v) in phosphate buffered saline (PBS, pH 7.2), divided into minimal use aliquots, and stored at −80 °C until use. Frozen animal cell culture lysates of poliovirus 1, feline calicivirus, Tulane virus, and hepatitis A virus that are routinely used in our laboratory were used in exclusivity studies. Adenovirus 41 strain Tak (VR-930D), bacteriophage MS2 (15597-B1), *Escherichia coli* [(Migula) Castellani and Chalmers strain C3000 (15597)], and *Enterobacter cloacae* subsp. Cloacae [(Jordan) Hormaeche and Edwards (13047)] obtained from ATCC (Manassas, VA) were also used. For those studies, genomic nucleic acids from all samples were extracted using the NucliSENS^®^ easyMAG system (bioMerieux SA, Marcy l′Etoile, France) according to the manufacturer’s instructions. All extracted nucleic acids were aliquoted into minimal use volumes and stored at −80 °C until use.

### Generation of a human norovirus RNA Standard by RT-qPCR

The amplifiable genomic copies of a sample was determined using an RNA standard curve as previously described^37^. Briefly, extracted GII.4 New Orleans RNA (Accession Number: JN595867) was first amplified with T7GII.4F and GII.4R primers (Table 1) using the Superscript One-Step RT-PCR kit (Invitrogen, Carlsbad, CA) and 5 μl template. A 15 min reverse transcription cycle at 50 °C was then followed by enzyme inactivation at 95 °C for 2 min. Amplification was performed for 30 cycles of 95 °C for 15 sec, 55 °C for 30 sec, and 72 °C for 30 sec. The 460 nucleotide amplicon was gel purified using the QIAquick gel extraction kit (Qiagen, Hilden, Germany) and subjected to *in vitro* transcription using the MEGAshortscript T7 kit (Thermo Fisher, Waltham, MA) according to manufacturer’s instructions. The transcribed RNA was purified and quantified (NanoPhotometer Pearl, Denville Scientific, South Plainfield, NJ). It was then serially diluted and used to construct a standard curve with RT-qPCR using the JJV2F-COG2R-Ring2-TP primer probe set (Table 1) and the SuperScript One-Step RT-PCR kit with 45 cycles and a 54 °C annealing temperature. The RNA standard curve was used to estimate RNA copy number from extracted patient stool samples. Cq was evaluated with a threshold of 30 flourescence units using Bio-Rad CFX Manager software. All reactions were performed in triplicate.

### Real time RT-RPA primer and probe design

Primers for RT-RPA were designed following manufacturer guidelines (TwistDx Inc. Cambridge, UK). Targets for primer design were the relatively conserved ORF1-2 junction and a previously reported conserved region of the norovirus capsid near the N/S domain of ORF2^15^,^38^. After initial screening, one optimally performing primer set was selected and the corresponding probe was designed according to manufacturer’s instructions (TwistDx Inc.) (Table 1). Primers were provided by Integrated DNA Technologies (IDT, Coralville, IA) and probes were provided by Biosearch Technologies (Novato, CA).

### RT-RPA reaction conditions

RT-RPA reactions were carried out using the TwistAmp exo RT kit (TwistDx Inc.). Each 50 μl reaction contained 2.1 pmol forward and reverse primer, 0.6 pmol probe, 29.5 μl proprietary rehydration buffer, 10 μl template, and nuclease-free water to 47.5 μl. The reaction mixture was then added to rehydrate TwistAmp exo RT lyophilized enzyme pellets, and 2.5 μl of 280 mM magnesium acetate was added to the tops of reaction tube lids. The magnesium acetate droplets were then spun down using a minifuge and a timer immediately started. Reactions were quickly transferred to a Bio-Rad CFX Thermal Cycler (Bio-Rad, Hercules, CA) set to 40 °C with cycles read every 30 sec. Five min after the reactions were started, the cycler was paused, reactions mixed, spun down, and placed back on the thermal cycler to continue cycling. The total time to produce signal was calculated by the following equation (1): (Total Time Thermal Cycler Not Recording Reaction) + (Ct Value * 30 sec/cycle).

### Evaluation of purified RNA and heated stool samples using RT-RPA and RT-qPCR

Twenty percent suspensions of norovirus GII.4 New Orleans-confirmed clinical samples from 12 patients were serially diluted and the RNA released by heating in a Bio-Rad T100 Thermal Cycler (Bio-Rad, Hercules, CA) at 99 °C for 5 min. Tubes were cooled on ice, mixed and briefly centrifuged. These heated stool dilutions were used directly as a template in RT-RPA reactions as described above, and in RT-qPCR reactions using the JJV2F-COG2R-Ring2-TP primer probe set (Table 1). In addition, NucliSENS^®^ easyMAG RNA extracts from each clinical sample were serially diluted and used in RT-RPA and RT-qPCR. The degree of correlation between RT-RPA time to detection (in min) and log_10_ genomic copy input (by standard curve) was determined by linear regression using Microsoft Excel 2013 (Figure S2). For evaluating the RT-RPA assay analytical sensitivity, serial dilutions of purified RNA from three selected stool samples (samples 29, 47, and 58) were tested using as described above for a total of 8 replicates and a probit regression was performed for each using the JMP 12 software package (SAS, Cary, NC). The limit of detection and standard deviation was calculated by averaging the three values produced by the regressions. Each value reflected the predicted LGC for which 95% of tests would produce a positive result. All reaction sets included no template controls to confirm lack of contamination.

### Exclusivity testing and sequence analysis

The specificity was evaluated by analyzing multiple relevant enteric virus and bacterial strains for cross reactivity with the RT-RPA assay (Table 3). Purified genomic nucleic acid was serially diluted, and 10^−2^ and 10^−3^ dilutions used directly as templates in RT-RPA. To analyze the degree of sequence difference between GII.4 New Orleans, for which the primers were designed, and two other human norovirus strains (GII.3 and GII.4 Sydney), sequences were aligned by performing Clustal W analysis using the MEGA 6 Software suite^39^.

## Additional Information

**How to cite this article**: Moore, M. D. and Jaykus, L.-A. Development of a Recombinase Polymerase Amplification Assay for Detection of Epidemic Human Noroviruses. *Sci. Rep.* **7**, 40244; doi: 10.1038/srep40244 (2017).

**Publisher's note:** Springer Nature remains neutral with regard to jurisdictional claims in published maps and institutional affiliations.

## Supplementary Material

Supplemental Figures

## Figures and Tables

**Table 1:** Primer sequences used for RT-RPA and RT-qPCR assays.

Primer Name	Sequence (5′ → 3′)	Nucleotide^a^	Source
JJV2F^b^	CAAGAGTCAATGTTTAGGTGGATGAG	5003	Jothikumar *et al*.^14^
COG2R^b^	TCGACGCCATCTTCATTCACA	5101	Jothikumar *et al*.^14^
Ring2-TP^b^	TGGGAGGGCGATCGCAATCT	5048	Jothikumar *et al*.^14^
NOF5	CCACGGCCCAGCATTTTACAGCAAAATCAGC	4918	This Paper
NOF7	CCATACAATTGATGTCCCTACTGGGGGAGGCCGC	4880	This Paper
NOR9	TTCTAGGGGATACTGTAAACTCTCCACCAGGGGC	5292	This Paper
G2R10	CCTGGGGCATTTCTAGGGGATACTGTAAACTCTCC	5304	This Paper
NOR11	CTACGGGCTCCAAAGCCATAACCTCATTGTTGACC	5182	This Paper
NOR12	CCAAAGCCATAACCTCATTGTTGACCTCTGGG	5172	This Paper
NOP1^d^	ATTTTTACGTGCCCAGACAAGAGCCAATGT3CAHA1GGATGAGATTCTCAGA	4987	This Paper
T7GII.4F^c^	TAATACGACTCAACTATAGCAAGAGTCAATGTTTAGGTGGATGAG	5003	This Paper
GII.4R2^c^	GTTGGGAAATTCGGTGGGACTG	5182	This Paper

RT-PCR and RT-qPCR reactions were both cycled at a 15 min reverse transcription cycle at 50 °C, followed by reverse transcriptase inactivation at 95 °C for 2 min, then amplification for 30 or 45 cycles of 95 °C for 15 sec, 55 °C or 54 °C for 30 sec, and 72 °C for 30 sec, respectively. Primer and probe reaction concentrations were all 200 nM.

^a^Nucleotide corresponding to 5′ of primer on GII.4 New Orleans sequence (GenBank JN595867.1).

^b^54 °C annealing temperature and 2.1 pmol primer in 50 μl reaction used for RT-qPCR primers and probe.

^c^55 °C annealing temperature and 2.1 pmol primer in 50 μl reaction used for RT-PCR amplification of standard.

^d^For probe modifications: 3 = internal dT-FAM; H = THF; 1 = internal dT-BHQ1. Probe has 3′ C3-spacer for blocking extension.

**Table 2:** RT-RPA performance with purified RNA and heated stool samples.

Isolate Number	Purified GII.4 New Orleans RNA			Heated GII.4 New Orleans Stool		
	Input (LGC)^a^	Time to Detection (Min)	Lowest Detectable Input (LGC)^b^	Input (LGC)^a^	Time to Detection (Min)	Lowest Detectable Input (LGC)^b^
10	1.6–7.6	6.1 ± 0.1–13.2 ± 2.8	4.6 (4/4)	2.2–8.2	7.3 ± 0.0^c^–15.4 ± 0.5	5.2 (2/3)
14	3.4–9.4	6.8 ± 0.3–14.8 ± 3.0	4.4 (1/3)	4.0–10.0	6.7 ± 1.2–16.7 ± 1.4	5.0 (2/3)
15	2.0–8.0	7.3 ± 0.8–14.0 ± 0.0^c^	5.0 (1/3)	2.6–8.6	7.9 ± 1.7–14.7 ± 0.0^c^	4.6 (1/3)
29	0.2–6.2	10.4 ± 1.7–13.3 ± 0.2	4.2 (3/3)	0.8–6.8	11.2 ± 0.1–14.1 ± 0.0^c^	2.8 (1/3)
34	3.0–9.0	7.4 ± 0.5–13.4 ± 2.6	5.0 (3/3)	3.6–9.6	7.8 ± 1.5–11.8 ± 0.0^c^	6.6 (1/3)
37	1.3–7.3	9.3 ± 0.9–14.2 ± 2.2	5.3 (3/3)	1.9–7.9	8.4 ± 0.3–10.9 ± 2.8	5.9 (3/3)
44	0.5–6.5	11.7 ± 0.0^c^–15.3 ± 3.5	3.5 (1/3)	1.1–7.1	None Detected	None Detected
47	0.2–6.2	9.5 ± 0.5–21.5 ± 0.0^c^	3.2 (1/3)	0.8–6.8	19.8 ± 1.2	6.8 (3/3)
58	0.6–6.6	7.6 ± 0.2–19.6 ± 0.0^c^	1.6 (1/3)	1.2–7.2	9.4 ± 1.7–11.3 ± 1.0	5.19 (3/3)
64	0.7–6.7	9.3 ± 0.4–14.1 ± 2.7	4.7 (3/3)	1.3–7.3	18.2 ± 0.0^c^	5.3 (1/3)
74	2.5–8.5	8.1 ± 0.6–13.2 ± 2.2	4.5 (3/3)	3.1–9.1	12.8 ± 0.3–20.0 ± 0.6	7.1 (3/3)
87	3.0–9.0	6.8 ± 0.1–13.1 ± 1.2	5.0 (3/3)	3.6–9.6	7.5 ± 0.1–9.9 ± 0.9	7.6 (3/3)

Twelve outbreak stool specimens previously confirmed positive for GII.4 New Orleans were diluted 20% in PBS and either subjected to genomic RNA purification with serial dilution or serially diluted and boiled at 99 °C.

^a^The input range tested for each sample.

^b^The lowest input level for which a signal was obtained along with the proportion of replicates the signal was obtained for that input level.

^c^No standard deviation observed due to only one positive replicate.

**Table 3:** Exclusivity (specificity) analysis.

Organism Type^a^	Organism name	Signal (Y/N)^b^
Virus	Human norovirus GII.4 Sydney strain	Y (4/4)
Virus	Human norovirus GI.6	N
Virus	Human norovirus GII.3	Y (1/3)
Virus	Poliovirus 1	N
Virus	Feline calicivirus	N
Virus	Tulane virus	N
Virus	Adenovirus 41 strain Tak (ATCC VR-930D)	N
Virus	Hepatitis A virus	N
Virus	Bacteriophage MS2 (ATCC 15597-B1)	N
Enteric bacteria	*Escherichia coli* (Migula) Castellani and Chalmers Strain C3000 (ATCC 15597)	N
Enteric bacteria	*Escherichia coli* O157:H7	N
Enteric bacteria	*Enterobacter cloacae* subsp. Cloacae (Jordan) Hormaeche and Edwards, subsp. nov. (ATCC 13047)	N

The genomic RNA/DNA of several enteric viruses and bacteria was extracted using a NucliSens EasyMAG (bioMerieux), and 10^−2^ and 10^−3^ dilutions of the extracts were loaded as template in the RT-RPA assay using the G2F5-G2R11-G2P1 primer-probe set.

^c^Showed low reactivity in one replicate.

^a^Source organism type used. All organisms may be present in human enteric samples.

^b^Whether or not an amplifiable signal was observed at any point for any dilution with RT-RPA. If yes, then the proportion of replicates for which a positive signal was obtained is presented in parentheses.

**Table 4:**
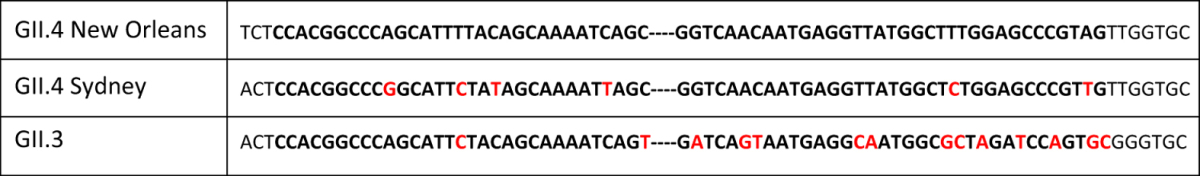
Sequence alignment of GII.4 strains and GII.3.

The sequences of GII.4 New Orleans and Sydney strains as well as a GII.3 strain were aligned using the Mega 6 software package. Bolded sequences correspond to the NOF5 and NOR11 target sequence regions, with base mismatches for Sydney and GII.3 colored red. Probe target regions were excluded because there were 0 and 3 mismatches with New Orleans as compared to Sydney and GII.3, respectively.
