# Supplementary material for: Development of a Recombinase Polymerase Amplification Assay for Detection of Epidemic Human Noroviruses
Source: Sci Rep. 2017 Jan 9;7:40244. doi: 10.1038/srep40244 (PMC5220337; doi:10.1038/srep40244)
Supplement: Supplemental Figures [file srep40244-s1.pdf]

Development of a Recombinase Polymerase Amplification Assay for Detection of Epidemic Human Noroviruses

Matthew D. Moore and Lee-Ann Jaykus

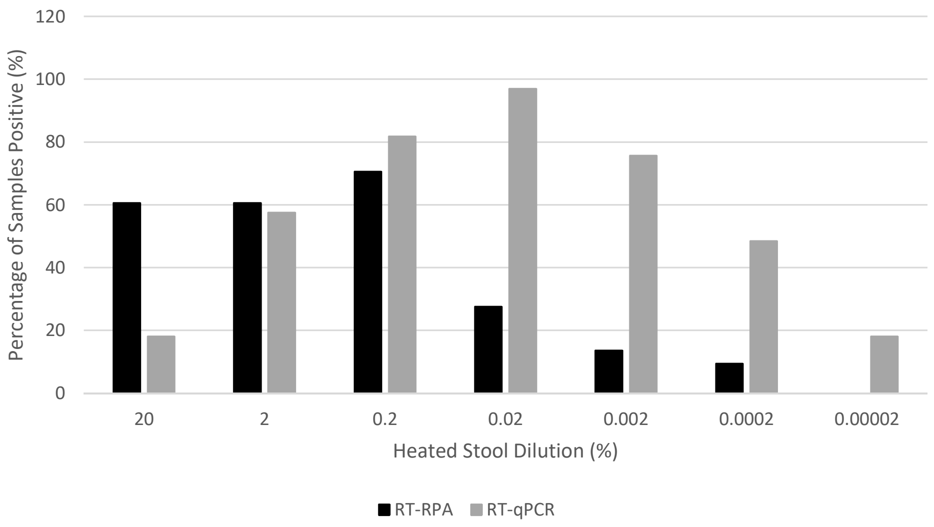

Figure S1. Comparison of RT-RPA and RT-qPCR assays for detection of heated stool isolates. Stocks of a 20% solutions of 12 clinical stool isolates were diluted and heated at 99°C for 5 min to release norovirus RNA. The heated samples were cooled and used directly as templates in RT-RPA or RT-qPCR reactions. The overall percentage of replicates at each stool dilution for all 12 isolates that produced a positive signal are presented in the figure. (*1.5 Column Image*)

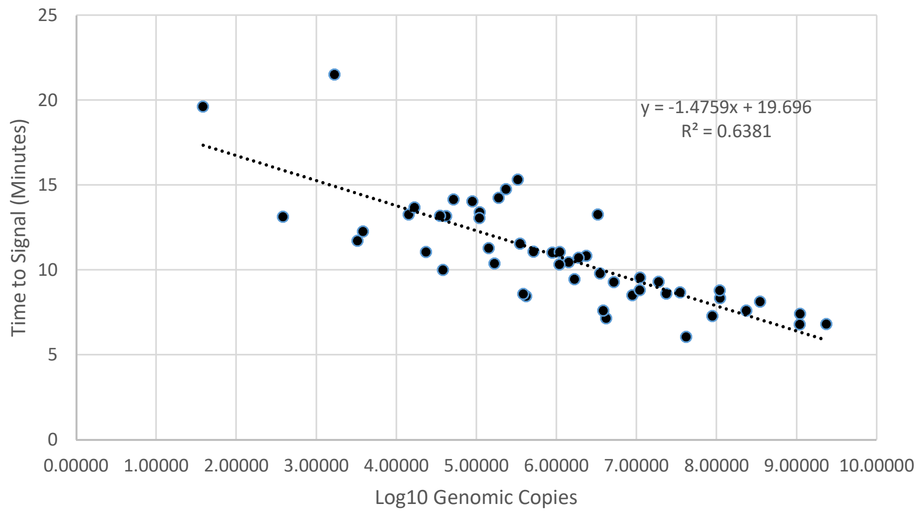

Figure S2. Linear Regression of RT-RPA assay with purified RNA. The time-to-fluorescence observed using the RT-RPA was plotted relative to the input log_{10} genomic copies for each reaction in Microsoft Excel 2013. A line of best fit was then plotted and equation and R^2 are displayed.

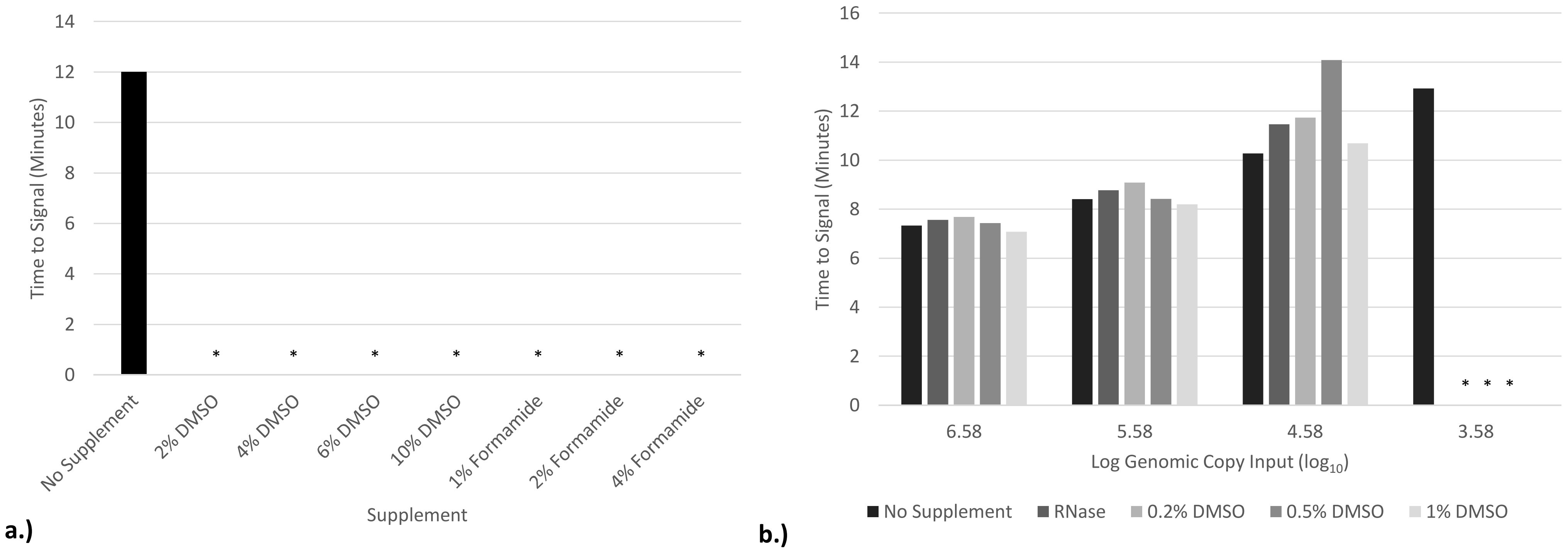

Figure S3. Effect of RNase, DMSO, and Formamide on RT-RPA assay. The effects of different supplements to the RT-RPA master mix that traditionally may improve amplification reactions were investigated. a.) Effects of DMSO and formamide on improving limit of detection of RT-RPA assay. A low concentration of human norovirus RNA approaching the limit of detection of the assay, 3.55 LGC, was added to RT-RPA reactions containing different degrees of DMSO or formamide. b.) Effects of RNase, DMSO, and formamide on time to signal. Different dilutions of the same human norovirus RNA were tested for different master mix formulations containing either 20 U RNasein RNase Inhibitor (Promega), 0.2% DMSO, 0.5% DMSO, 1% DMSO, or no supplement. For both * indicates that there was no detectable signal (negative result).

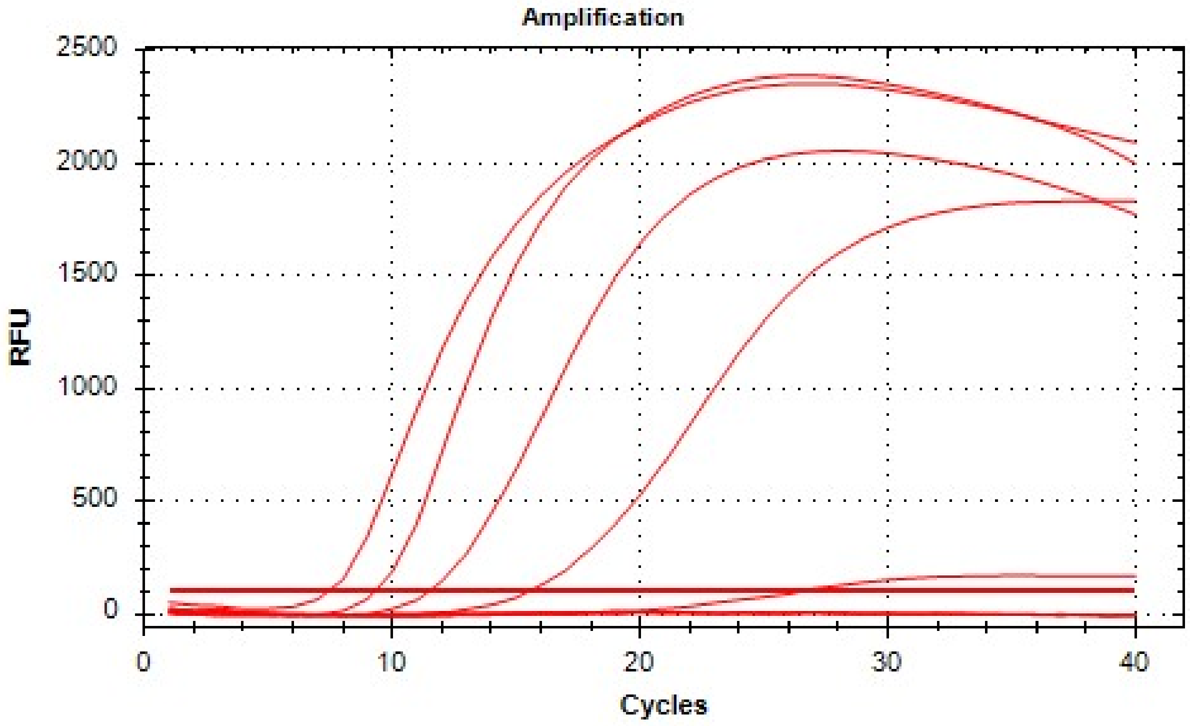

a.)

b.)

Log Genomic Copies (log_{10})	Ct	Time (Minutes)
9.25	7.81	6.91
8.25	9.58	7.79
7.25	11.89	8.94
6.25	16.13	11.06
5.25	28.73	17.37

Figure S4. Representative RT-RPA Dilution Curve. An example dilution curve for the RT-RPA assay is pictured. The raw data curve readout a.) is shown as visualized using the Bio-Rad CFX Manager 3.1 software (Bio-Rad). The associated data obtained from the curve is provided in table format in b.).
